# Transferability of health cost evaluation across locations in oncology: cluster and principal component analysis as an explorative tool

**DOI:** 10.1186/s12913-014-0537-x

**Published:** 2014-11-18

**Authors:** Lionel Perrier, Alessandra Buja, Giuseppe Mastrangelo, Patrick Sylvestre Baron, Françoise Ducimetière, Petrus J Pauwels, Carlo Riccardo Rossi, François Noël Gilly, Amaury Martin, Bertrand Favier, Fadila Farsi, Mathieu Laramas, Vincenzo Baldo, Olivier Collard, Dominic Cellier, Jean-Yves Blay, Isabelle Ray-Coquard

**Affiliations:** University of Lyon, F-69007 Lyon, CNRS, GATE Lyon-St Etienne, UMR 5824 Ecully, France; Clinical Research and Innovation Direction (DRCI), Cancer Centre Léon Bérard, 28 rue Laënnec, 69008 Lyon, France; Department of Molecular Medicine, Laboratory of Public Health and Populations Studies, University of Padova, 35122 Padova, Italy; Department of Cardiac Thoracic and Vascular Sciences, University of Padova, 35122 Padova, Italy; Economics Department, University of Lyon, 69007 Lyon, France; Cancer Centre Léon Bérard, INSERM EAM 4128 Santé-Individu-Société, University of Lyon, 28 rue Laënnec, 69373 Lyon Cedex 08, France; Cancéropôle Lyon Auvergne Rhône-Alpes, 60 avenue Rockefeller, 69008 Lyon, France; Melanoma and Sarcomas Unit, Veneto Institute of Oncology, IOV, IRCCS, University of Padova, 35128 Padova, Italy; University of Lyon, Department of Digestive Surgery, University Hospital Lyon Sud, 165 Chemin du Grand Revoyet, 69310 Pierre Bénite, France; Pharmacy, Cancer Centre Léon Bérard, 28 rue Laënnec, 69373 Lyon Cedex 08, France; Réseau Espace Santé Cancer, Rhône-Alpes, 60 avenue Rockefeller, 69008 Lyon, France; Department of Medical Oncology, University Hospital of Grenoble, BP 217 38043 Grenoble cedex 09, France; Department of Medical Oncology, Institut de Cancérologie Lucien Neuwirth, 108 B Avenue Albert Raimond, 42270 Saint-Priest-en-Jarez, France; Department Cancer and Environnement, Cancer Centre Léon Bérard, 28 rue Laennec, 69373 Lyon Cedex 08, France; University of Lyon, Department of Medical Oncology, Cancer Centre Léon Bérard, 28 rue Laennec, 69373 Lyon Cedex 08, France

**Keywords:** Cluster analysis, Cancer network, Cost, Economic evaluation, Oncology, Principal component analysis, Sarcoma, Transferability, Variability

## Abstract

**Background:**

The transferability of economic evaluation in health care is of increasing interest in today’s globalized environment. Here, we propose a methodology for assessing the variability of data elements in cost evaluations in oncology. This method was tested in the context of the European Network of Excellence “Connective Tissues Cancers Network”.

**Methods:**

Using a database that was previously aimed at exploring sarcoma management practices in Rhône-Alpes (France) and Veneto (Italy), we developed a model to assess the transferability of health cost evaluation across different locations. A nested data structure with 60 final factors of variability (e.g., unit cost of chest radiograph) within 16 variability areas (e.g., unit cost of imaging) within 12 objects (e.g., diagnoses) was produced in Italy and France, separately. Distances between objects were measured by Euclidean distance, Mahalanobis distance, and city-block metric. A hierarchical structure using cluster analysis (CA) was constructed. The objects were also represented by their projections and area of variability through correlation studies using principal component analysis (PCA). Finally, a hierarchical clustering based on principal components was performed.

**Results:**

CA suggested four clusters of objects: chemotherapy in France; follow-up with relapse in Italy; diagnosis, surgery, radiotherapy, chemotherapy, and follow-up without relapse in Italy; and diagnosis, surgery, and follow-up with or without relapse in France. The variability between clusters was high, suggesting a lower transferability of results. Also, PCA showed a high variability (i.e. lower transferability) for diagnosis between both countries with regard to the quantities and unit costs of biopsies.

**Conclusion:**

CA and PCA were found to be useful for assessing the variability of cost evaluations across countries. In future studies, regression methods could be applied after these methods to elucidate the determinants of the differences found in these analyses.

**Electronic supplementary material:**

The online version of this article (doi:10.1186/s12913-014-0537-x) contains supplementary material, which is available to authorized users.

## Background

Economic evaluations have become an integral part of healthcare decision-making worldwide; however, cost assessments are time consuming, expensive, and not systematically reproducible. The value of these studies, according to Nixon, is determined by the methods used and transparency in reporting [1]. Although the number of economic evaluations of pharmacoeconomic guidelines has increased, the use of economic evaluations in other jurisdictions usually requires the implementation of methodological adaptations for the specific environment under investigation [2,3].

Sculpher et al. suggested that the generalizability of economic evaluations is based on the extent to which results from a study of a particular patient population and/or specific context can be transposed to another population and/or a different context [4]. Alternatively, transferability represents the ability to substitute local data with data from other environments, allowing the analysis to be easily applied to other settings or countries [1]. Generally, the data used in economic evaluations include pricing (unit costs of resources used and quantities) and clinical practices (characteristics of disease and corresponding procedures of diagnosis, treatment, and follow-up).

After the pioneering work of Drummond [5], many authors have employed a qualitative approach, which is based on systems, tools, checklists, and flow charts, in order to assess or guide transferability practices during economic evaluation. This topic has been recently reviewed by Goeree [6].

Quantitative methods, mainly based on regression modeling, have been largely used to explain variability in costs and/or cost-effectiveness by location [7-15]. More specifically, multilevel regression models were employed to analyze data that fall naturally into hierarchical structures, consisting of multiple macro units (countries) and multiple micro units (centers) within each macro unit [4]. We aim to achieve an overall picture of things, which could make it possible to focus on the main problems and identify hypotheses using cluster analysis (CA) and principal component analysis (PCA). CA and PCA are examples of how distances and the assumption of correlations among numerous quantitative variables can be used to display whether the phenomena are near or far in a simple plot. Regression methods could be applied after such explorative analyses to recognize the determinants of the differences found.

To our knowledge, only one abstract using CA to explore the transferability of cost assessment among countries has been published [16], while none have utilized PCA. In the present study we have used both methods, CA and PCA, to assess the variability of health care costs in sarcoma management in two European regions, Rhône-Alpes (France) and Veneto (Italy). The rarity of these tumors and the large variability in their clinical and histopathological presentation makes the standardization of therapeutic sequences difficult, making this research particularly important with respect to transferability of its economic evaluation [17].

The objective of the research was then to ascertain the contributions of various stages of cancer care, more specifically their unit costs and resource use to between country differences.

## Methods

Our initial cohort consisted of 327 sarcoma patients who were ≥15 years-old (254 in Veneto, Italy and 73 in Rhône-Alpes, France). All patients had histological confirmation of primary malignant sarcoma, with or without distal metastases at initial diagnosis. All patients from Rhône-Alpes (n = 73) had been diagnosed between March 2005 and February 2006 and were recruited from two sites (the University Hospital of Lyon and the Léon Bérard Cancer Centre). The patients from Veneto (n = 161) were diagnosed between January 2007 and December 2007 and recruited at one of the 22 public hospitals in the region. Absence of patient consent (n = 55), care undertaken outside the participating regions or in private hospitals (n = 23), and missing records (n = 30) reduced the number of patients included in the study to 219, 58 from Rhône-Alpes and 161 from Veneto [17]. These patients were followed retrospectively using prospectively implemented databases for three years after their initial sarcoma diagnosis or until death. In addition, patients were managed in accordance with the ethical principles for medical research involving human subjects described in the Declaration of Helsinki. The cost evaluation of the 219 sarcoma patients began in 2009. For each patient, quantities of resources used (number of days in hospital, cycles and doses of chemotherapy, number of transfusions, radiotherapy sessions, imaging procedures, biopsies, surgical process and consultations) were collected for each sequences of management (i.e. initial diagnosis, initial surgery, chemotherapy, radiotherapy, follow-up with relapsed patients, and follow-up with healthy patients). Costs were assessed from the hospital’s perspective from the time of diagnosis to the end of follow-up (or death), designating the country (France or Italy) and the sequence of management. Average unit costs were assessed for France and Italy respectively and applied in respect to the patient management country of origin. All costs were expressed in 2009 euro. 4% discounting per year was applied according to the French Health Authority’s recommendation to both countries [18]. The study received approval in France from the National Ethics Committee (N°904073) and the National Committee for Protection of Personal Data (N°05-1102), and from the Local Sanitary Agency of the Veneto Region and the Ethics Committee of the Azienda Ospedaliera di Padova (N°156/06/CE) in Italy. Data were collected within the context of the European network of excellence “Connective Tissues Cancers Network” (CONTICANET, FP6 018806), which is funded by the European Commission. The full protocol of the project has been previously published [19,20].

### Definition

Welte and Sculpher suggested that, in order to clearly structure the transferability assessment, it is necessary to systematically identify the factors that impact the variability (final factors) and to gather them into homogeneous categories (areas of variability) [21,4]. Accordantly to model the problem of assessing transferability of health cost evaluation across locations we identified 60 final factors of variability (e.g. unit cost of chest radiograph). In addition to fit better our data regarding entire management process of a sarcoma disease we applied a nested structure within 16 variability areas (e.g. unit cost of imaging) and within 12 objects (e.g. diagnoses) in Italy and France, independently.

#### Identification of potential and final factors of variability

A factor is a potential source of variability in the relative prices and quantities (i.e. unit cost, number of surgical biopsies, radiotherapy sessions, etc.). They were identified from the literature [1,4-6,22-24]. Only those factors that varied from one to another country were included in the analysis (final factors), i.e. factors that do not vary with geography were not included.

#### Area of variability

Each area of variability included a set of final factors. The complete list of final factors within these areas is reported in Table 1. An example of an area is the “area quantity of imaging”, which included five final factors: “chest radiograph”, “colonoscopy”, “computed tomography”, “ultrasound”, and “magnetic resonance imaging”. Since each final factor can vary according to “unit cost” and “number of resources used”, a total of 16 (8 × 2 = 16) areas of variability were generated (Table 1). For example, imaging was classified in Area 3 considering resources used and Area 11 considering costs.

**Table 1:** Final factors included in the construction of the 16 areas of variability and method of calculation of factors inside each area

**Area (Source of information)**	**Final factors of variability included**	**Methods of calculation**
Area 1	Number of: surgical biopsies, micro-biopsies, needle	Sum of the factors per patient
Q. biopsies (A)	aspiration cytology	Calculation of the mean per object
Area 2	Number of: days for inpatient; days for outpatient	Sum of the factors per patient
Q. hospital admissions (A)		Calculation of the mean per object
Area 3	Number of: chest radiograph, colonoscopy, computed tomography, ultrasound, magnetic resonance imaging	Sum of the factors per patient
Q. imaging (A)		Calculation of the mean per object
Area 4	Number of external consultations	Calculation of the mean per object
Q. external consultations (A)		
Area 5	Number of: Ps packs, red blood cell packs	Sum of the factors per patient
Q. transfusions (A)		Calculation of the mean per object
Area 6	Number of radiotherapy sessions	Calculation of the mean per object
Q. radiotherapy (A)		
Area 7	Number of preparations for radiotherapy sessions	Calculation of the mean per object
Q. preparation for radiotherapy (A)		
Area 8	Drug administration (yes/no): Caelyx, Carboplatin, Cisplatin, Deticene, Doxorubicin, Etoposide, Gemcitabin, Holoxan, Ifosfamide, Imatinib, Melphalan, Vinorelbin, Oxaliplatin, Paclitaxel, Vincristine	Sum of the “yes” for the fifteen factors per patient.
Q. chemotherapy drugs (A)		
		Calculation of the mean per object
Area 9	Unit cost of: surgical biopsies; microbiopsies; needle	Calculation of the mean cost of the factors by country.
C. biopsies (B, C)	aspiration cytology	Application of the mean (by country) to the objects with quantity of biopsies >0
Area 10	Unit cost of: days for inpatient, days for outpatient	Calculation of the mean of the factors by country.
C. hospital admissions (D)		Application of the mean (by country) to the objects with quantity of days of hospital admissions >0
Area 11	Unit cost of: chest radiograph; colonoscopy; computed tomography; ultrasound; magnetic resonance imaging	Calculation of the mean of the factors by country.
		Application of the mean (by country) to the objects with quantity of imaging >0
C. imaging (B, C)		
Area 12	Unit cost of external consultations	Application (distinguishing the country) to the objects with quantity of external consultations >0
C. external consultations (B,C)		
Area 13	Unit cost of: Ps packs, red blood cell packs	Calculation of the mean of the factors by country.
C. transfusions (E,C)		Application of the mean (by country) to the objects with quantity of transfusion packs >0
Area 14	Unit cost of radiotherapy sessions	Application (distinguishing the country) to the objects with quantity of radiotherapy sessions >0
C. radiotherapy (B,C)		
Area 15	Unit cost of preparation for radiotherapy sessions	Application (by country) to the objects with quantity of preparation for radiotherapy sessions >0
C. Preparation for radiotherapy (B,C)		
Area 16	Unit cost of: Caelix; Carboplatin; Cisplatin; Deticene; Doxorubicin; Etopophos etoposide; Gemcitabine; Holoxan; Ifosfamide; Imatinib; Melphalan; Navelbine; Oxaliplatin; Paclitaxel; Vincristine	Calculation of the mean of the factors by country.
C. Chemotherapy drugs (F)		Application of the mean (by country) to the objects with quantity of chemotherapy drugs >0

Sources of information: A = Medical records; B = Classification commune des actes médicaux (France); C = Regione del Veneto, Nomenclatore tariffario prestazioni specia-listiche ambulatoriali (Italy); D = Hospital Managers; E = Arrêté du 2 janvier 2008 relatif au tarif de cession des produits sanguins labiles JORF, 10 février 2008, n°35 (France); F = Hospital pharmacists; Q. = quantity; C. = unit cost.

Formally, the values of the *n =* 16 areas of variability for *m* = 12 objects give a matrix **A**_(*mxn*)_ with the general term α_*ij*_ with α_*ij*_ ≥0.

$$ {\mathbf{A}}_{\left(m\times n\right)}=\left[{\alpha}_{ij}\right],\kern0.5em {\alpha}_{ij}\ge 0 $$

To overcome variability due to differences in monetary units and/or quantity of health resources used, all of the variables were standardized, passing from the matrix **A**_(*mxn*)_ = [α_*ij*_] to the matrix **X**_(*mxn*)_ = [*x*_*ij*_] where:

$$ x{}_{ij}=\frac{\alpha_{ij}-{\overline{\alpha}}_j}{\sigma_j}\kern1em {\overline{\alpha}}_j=\frac{1}{m}{\displaystyle \sum_{i=1}^m{\alpha}_{ij}\kern1em }{\sigma}_j=\sqrt{\frac{1}{m}{\displaystyle \sum_{i=1}^m{\left({\alpha}_{ij}-{\overline{\alpha}}_j\right)}^2\kern1em }} $$

#### Object

Six phases of management (diagnosis, surgery, chemotherapy, radiotherapy, follow-up with relapsed patients, and follow-up with healthy patients) and two countries (Italy and France) were delineated. We therefore generated 12 objects (6 × 2 = 12), which included: diagnosis in France (object 1); diagnosis in Italy (object 2); surgery in France (object 3); surgery in Italy (object 4); chemotherapy in France (object 5); chemotherapy in Italy (object 6); radiotherapy in France (object 7); radiotherapy in Italy (object 8); follow-up without relapse in France (object 9); follow-up without relapse in Italy (object 10); follow-up with relapse in France (object 11); and follow-up with relapse in Italy (object 12).

### Statistical analysis

An individual patient level dataset was completed accounting for resources consumed during the defined time period of the study, broken down into different phases of care (objects) and different areas (e.g. quantity of biopsies) per country and also accounting for the average unit cost of resource per country.

#### Cluster analysis

Cluster analysis involves assigning a set of objects into groups (called clusters) so that the objects in the same cluster are more similar to each other than to those in other clusters. Clustering required three steps in order to define the following parameters: distances, hierarchical structure, and optimal number of clusters [25].

First, the distances between all the pairs of objects can be evaluated using Euclidean distance, Mahalanobis distance, and city-block metric [26]. Given an *m*-by-*n* data matrix **X**, which is treated as m (1-by-n) row vectors ***x***_1_,***x***_2_,…,***x***_*m*_, the various distances between the vector ***x***_*r*_ and ***x***_*s*_ are defined as follows:

*(i) Euclidean distance*: $$ {d}_{rs}^2=\left({x}_r-{x}_s\right)\kern0.5em {\left({x}_r-{x}_s\right)}^{\prime } $$

Due to the preceding normalization this is in fact a ‘Standardized Euclidean distance’.

(ii) Mahalanobis distance: ${d}_{rs}^2={\left({x}_r-{x}_s\right)}^{\prime }{V}^{-1}\left({x}_r-{x}_s\right)$ where ***V*** is the sample covariance matrix.

*(iii) City Block metric*: ${d}_{rs}={\displaystyle \sum_{j=1}^n\left|{x}_{rj}-{x}_{sj}\right|}$

However, when we used linkage procedures in a second stage, not all distances were relevant. Therefore, in order to make the best choice, we had to use the cophenetic correlation coefficient (see below).

Secondly, an iterative process (agglomerative hierarchical approach) was used to set a hierarchical structure. We put the distance information and link pairs of objects that were close together into binary clusters (made up of two objects). Then, these newly formed clusters were linked into larger clusters until all objects were linked together in a hierarchical tree. The hierarchical tree created by the linkage function was most easily understood when viewed graphically [27]. Therefore, we plotted this hierarchical information as a graph. The criteria used to compute distances between groups of objects were:

- Single linkage: minimum distance criteria, using the smallest distance between objects in the two groups:

$$ d\left(r,s\right)= \min \left( dist\left({x}_{ri},{x}_{sj}\right)\right)\kern1.5em i\in \left(1,\dots, {n}_r\right)\kern1em j\in \left(1,\dots, {n}_s\right); $$

- Complete linkage: maximum distance criteria, using the largest distance between objects in the two groups:

$$ d\left(r,s\right)= \max \left( dist\left({x}_{ri},{x}_{sj}\right)\right)\kern1.5em i\in \left(1,\dots, {n}_r\right)\kern1em j\in \left(1,\dots, {n}_s\right); $$

- Average linkage: using the average distance between all pairs of objects in cluster r and cluster s:

$$ d\left(r,s\right)=\frac{1}{n_r\times {n}_s}{\displaystyle \sum_{i=1}^{n_r}}{\displaystyle \sum_{j=1}^{n_s}} dist\left({x}_{ri},{x}_{sj}\right); $$

- Centroid linkage: using the distance between the centroids of the two groups:

$$ d\left(r,s\right)=d\left({\overline{x}}_r,{\overline{x}}_s\right)\kern1em {\overline{x}}_r=\frac{1}{n_r}{\displaystyle \sum_{i=1}^{n_r}{x}_{ri}\kern1em }{\overline{x}}_s=\frac{1}{n_s}{\displaystyle \sum_{j=1}^{n_s}{x}_{sj}}\kern1em ; $$

- Ward linkage: using the incremental sum of squares (i.e. the increase in the total within-group sum of squares as a result of joining groups *r* and *s*). It is given by:

$$ d\left(r,s\right)=\frac{n_r\times {n}_s}{n_r+{n}_s}{d}^2\left(r,s\right)=\frac{n_r\times {n}_s}{n_r+{n}_s}d\left({\overline{x}}_r,{\overline{x}}_s\right); $$

Where *d*^2^ (*r,s*) is the distance between cluster r and cluster s defined in the Centroid linkage. The within-group sum of squares of a cluster is defined as the sum of the squares of the distance between all objects in the cluster and the centroid of the cluster. The cophenetic correlation coefficient, as defined below, was used to select the most appropriate combination (i.e. metrics, linkage procedure).

For the final step of the clustering process, the clustering solution was evaluated by computing the cophenetic correlation coefficient “c”; the closer the coefficient value was to 1, the better the clustering solution. If **Y** gives distances computed in the step 1, and **Z** signifies distances generated by a linkage method in the step 2, then the cophenetic correlation coefficient between **Z** and **Y** was defined as:

$$ c=\frac{{\displaystyle {\sum}_{i<j}\left({Y}_{ij}-y\right)\left({Z}_{ij}-z\right)}}{\sqrt{{\displaystyle {\sum}_{i<j}{\left({Y}_{ij}-y\right)}^2{\displaystyle {\sum}_{i<j}{\left({Z}_{ij}-z\right)}^2}}}} $$

where:

*Y*_ij_ is the distance between objects *i* and *j* in *Y*.

*Z*_ij_ is the distance between objects i and j in *Z*.

*y* and *z* are the average of Y and Z, respectively.

The cophenetic correlation coefficient analyses are shown in Additional file 1. The best linkage method and the best distance are the pair (average; euclidean distance) with a cophenetic correlation coefficient equal to 0.83035.

A combination of good R-Square (RSQ) values was used to select the number of clusters to retain. More precisely, the optimum number of clusters to retain, which depends on homogeneity within cluster and/or heterogeneity between clusters, was assessed by RSQ, Semi-Partial R-Squared (SPRSQ), Root-Mean-Square Standard Deviation (RMSSTD) and the pseudo-F statistic (pF) [28,29]. Methods and measures used for determining the optimal number of clusters are:

- RSQ: RSQ measures the heterogeneity of the cluster solution formed at a given step. A large value represents that the clusters obtained at a given step are quite heterogeneous, whereas a small value signifies that the clusters formed at a given step are not very different from each other. It is therefore recommended to have a cluster solution with a high RSQ.

- SPRSQ: The SPRSQ measures the loss of homogeneity due to the merging of two clusters to form a new cluster at a given step. If the value is small, then it suggests that the cluster solution obtained at a given step is formed by merging two very homogeneous clusters. On the other hand, large values of SPRSQ suggest that two heterogeneous clusters have been merged to form the new cluster. In general, a cluster solution with a low SPRSQ is preferred, as a high value for SPRSQ implies that two heterogeneous clusters are being merged.

- RMSSTD: The RMSSTD measures the homogeneity of the cluster formed at any given step. It essentially measures the compactness or homogeneity of a cluster. Clusters in which consumers are very close to the centroid are compact clusters. The smaller the RMSSTD, the more homogeneous or compact the cluster formed is at a given step. A large RMSSTD value suggests that the cluster obtained at a given step is not homogeneous, and is probably formed by the merging of two heterogeneous clusters.

- pF: The pF is intended to capture the tightness of clusters, and is a ratio of the mean sum of squares between groups to the mean sum of squares within groups. It makes it possible to compare the homogeneity of a partition in k classes with that of a partition in (k-1) classes. A “strong” pF value at the level s will indicate a suitable partition in s classes correct. Peaks on the curve give the values of pF according to the number of classes.

**Figure 1:**
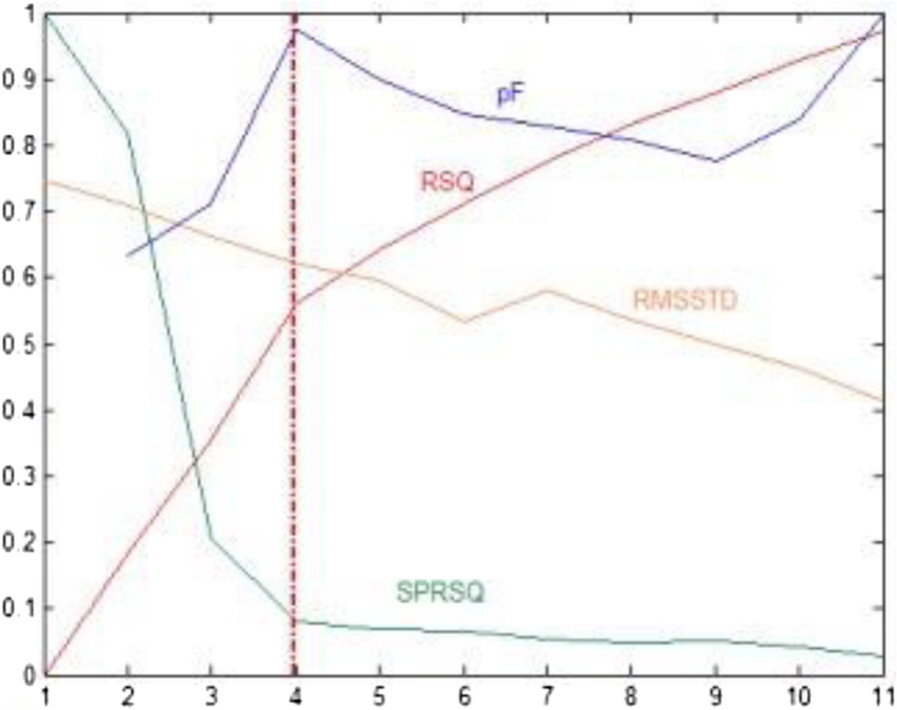
**Determination of optimal number of clusters.** pF: Pseudo-F statistic, RSQ: R-Square, RMSSTD: Root-mean-square standard deviation, SPRSQ: Semi-partial R-squared (pF has been scaled so that its values fall between 0 and 1, allowing it to be displayed in the same graph).

As recommended in the literature, all measures were used as they relate to various properties of the clusters (see Figure 1).

### Principal component analysis

To perform PCA, we began with the matrix **X**_(*m,n*)_, where *m* corresponded to the 12 objects (i.e. observations or individuals in statistical terms) and *n* to the 16 areas of variability (i.e. the variables). Individuals and variables did not have symmetrical roles therefore we had a different representation in the factorial plane (or hyper plane), with a different interpretation [30,31].

For **individuals,** we had *m* points that were located in the variable space **R**^*n*^. We then sought unit vectors **u**_α_ for defining a sub-space of **R**^*n*^ where in a projection of initial individuals-points was performed. Generally a projection is performed in the plane (**R**^2^). Vectors **u**_α_ were the eigenvectors of the matrix **X**^*T*^**X**, ranked in descending order of the corresponding eigenvalues. They were located on the factorial axes *F*_α._ The coordinates of the *m* individual points on the factorial axis *F*_α_ were the *m* components of the vector **ψ**_*α*_ = **Xu**_*α*_. The factor **ψ**_*α*_ was a linear combination of the initial variables. Individuals were described by coordinates denoted *ψ*_*α*_(*i*). They were associated with the measurements denoted CTR_α_(*i*) such that:

$$ CT{R}_{\alpha }(i)=\frac{\psi_{\alpha}^2(i)}{{\displaystyle {\varSigma}_i}{\psi}_{\alpha}^2(i)} $$

Individuals who made a strong contribution to the axis had a strong CTR_α_(*i*) (Additional file 2). Moreover, if a variable *x*_*j*_ was strongly correlated with, for example, *ψ*_1_ it meant that individuals with high positive (respectively negative) coordinates on axis 1 were characterized by a value of *x*_*j*_ well above (respectively below) the average (since the origin of axes of interest is the center of gravity of the cloud).

A projection of the objects in the first two components was made, and here we have added a three-dimensional graph because of the highest percentage of inertia of the first three components.

For **variables**, we had *n* points that were located in the space of individual **R**^*m*^. Each point was associated with a new point for which the coordinate on a factorial axis was a measurement of the correlation between the variable and the corresponding factor. In order to define a subspace of **R**^*m*^ we sought unit vectors **v**_α_. These were the eigenvectors of the matrix **XX**^*T*^ in decreasing order of corresponding eigenvalues. The coordinates of the variable points on the axis *α* were the *n* components of the vector **ϕ**_*α*_ = **X**^*T*^**v**_*α*_. We showed that the coordinate of a variable point on an axis was actually the correlation coefficient for this variable with the corresponding factor **ψ**_*α*_. Because the factorial axes were orthogonal pairs, we obtained a series of uncorrelated artificial variables called principal components, which synthesized the correlations of all the original variables.

In the space of dimension *m*, the distance between the point-variables and the origin was equal to 1, and therefore, by projection on a factorial plane, variables-points were part of a circle of radius 1, also known as the *circle of correlations.* These points were even closer to the edge of the circle as the variable point was well-represented by the factorial plane and thereby the variable was correlated with the two factors that made up the plane. Variables that were not located at the edge of the circle in a factorial plane were not correlated with the two corresponding factors and effectively were not useful for interpretation.

If we followed the first factorial plane, the coordinates of variable-points in the plane were given by quantities denoted *ϕ*_1_(*j*) and *ϕ*_2_(*j*). Considering the values of *ϕ*_*α*_(*j*) for the first two axes, the distance from the center of the circle, ${r}_j=\sqrt{\phi_1^2(j)+{\phi}_2^2(j)}$ was calculated and variables were sorted in descending order of *r*_*j*_.

Additional measures could be used to assist with the interpretation. In this regard, first the relative contribution of a variable to the inertia was explained by the axis *α*:

$$ CT{R}_{\alpha }(j)=\frac{\phi_{\alpha}^2(j)}{{\displaystyle {\varSigma}_j}{\phi}_{\alpha}^2(j)} $$

where *ϕ*_*α*_(*j*) represented the coordinate of the variable *j* on the axis *α* (Additional file 3) and second the quality of the representation of the variable *j* by its projection on the axis *α*:

$$ \cos {}_{\alpha}^2(j)=\frac{\phi_{\alpha}^2(j)}{\left\Vert {x}_j^2\right\Vert } $$

where ‖*x*_*j*_‖ was the norm (i.e. length) of the vector variable *j*.

#### ***Hierarchical clustering on principal component***

The simultaneous analysis of a principal component map and hierarchical clustering enriched the approach by representing the whole hierarchical tree in three dimensions on the principal component map [32], which was achieved by representing the centers of gravity of the partition (i.e. the highest nodes of the hierarchy).

Calculations were performed using MATLAB 6.1 (MathWorks, Inc. Natick, MA 01760 USA) and STATA 11 (StataCorp LP, College Station, TX 77845 USA).

## Results

The average costs of sarcoma management reached €26,156 (SD18,190) for patients diagnosed and treated in Rhône-Alpes (n = 58) and €24,986 (SD 24,575) for patients diagnosed and treated in Veneto (n = 161). The details of these mean costs of each stage of sarcoma management are shown in Table 2.

**Table 2:** Average costs (standard deviation) for each phase of sarcoma management by country (in €, 2009)

**Phases of treatment**	**All patients (n = 219)**	**Rhône-Alpes France (n = 58)**	**Veneto Italy (n = 161)**
Diagnosis	3,701 (7420)	1,392 (1,450)	4,534 (8,484)
Surgery (primary and wide surgical excision)	8,170 (7,364)	9,046 (6,226)	7,855 (7,749)
Chemo-therapy	6,107 (11,988)	9,689 (14,003)	4,815 (11,196)
Radio-therapy	2,270 (5,850)	1,615 (2,495)	2,505 (6,665)
Follow-up	5,048 (11,760)	4,414 (8,864)	5,277 (12,693)
Follow-up without relapse	1,468 (11,760)^a^	1,732 (1,728)^b^	1,379 (2,501)^c^
Follow-up with relapse	16,261 (11,760)^d^	11,454 (14,745)^e^	18,339 (21,611)^f^
Overall management	25,296 (22,919)	26,156 (18,190)	24,986 (24,575)

^a^n = 58; ^b^n = 42; ^c^n = 16; ^d^n = 161; ^e^n = 124; ^f^n = 37.

Table 3 reports data from Matrix **A**, which indicates the average of resources used and unit costs at the intersection of each column (16 areas of variability) and row (12 objects). In addition, the standardized data of Matrix **X** are reported in Additional file 4.

**Table 3:** Matrix A data: unit cost and quantity (final factors of variability) for 16 areas and 12 objects defined

**Objects**	**Areas of variability**															
	**Area 1 Q. Biopsies**	**Area 2 Q. days hospitalization**	**Area 3 Q. imaging**	**Area 4 Q. external consultations**	**Area 5 Q. transfusions**	**Area 6 Q. rad. sessions**	**Area 7 Q. rad. preparation**	**Area 8 Q. chemotherapy drugs**	**Area 9 C. biopsy**	**Area 10 C. day hospitalization**	**Area 11 C. imaging**	**Area 12 C. external consultation**	**Area 13 C. transfusion**	**Area 14 C. rad. session**	**Area 15 C. rad. preparation**	**Area 16 C. chemotherapy drugs**
1. Diagnosis France	1.34	1.29	3.66	0.76	0	0	0	0	59.5	918.3	76.34	90	0	0	0	0
2. Diagnosis Italy	2.04	5.4	4.61	1.19	0	0	0	0	41.63	602	76.24	47.45	0	0	0	0
3. Surgery France	0	13.38	1.98	0	0.8	0	0	0	0	918.3	75.59	0	199.6	0	0	0
4. Surgery Italy	0	10.53	2.37	0	1.72	0	0	0	0	602	76.24	0	26.3	0	0	0
5. Chemotherapy France	0	20.54	5.29	0	2.92	0	0	2.88	0	918.3	98.75	0	199.6	0	0	2.22
6. Chemotherapy Italy	0	18.36	8.06	0	1.52	0	0	2.03	0	602	67.16	0	26.3	0	0	2.98
7. Radiotherapy France	0	1.08	0	0	0	26.44	1	0	0	918.3	0	0	0	137	439	0
8. Radiotherapy Italy	0	11	0	0	0	19.87	1	0	0	602	0	0	0	150	871.8	0
9. Follow-up without relapse France	0	0.38	3.28	3.41	0	0	0	0.05	0	918.3	66.69	90	0	0	0	0.04
10. Follow-up without relapse Italy	0	1.01	5.29	4.01	0	0.02	0.01	0	0	602	76.24	47.45	0	150	871.8	0
11. Follow-up with relapse France	0	7.47	2.53	5.18	0	2.47	0.13	1.53	0	918.3	75.59	90	0	137	439	3.49
12. Follow-up with relapse Italy	0	20	11.41	5.06	0	0.26	0.04	0.47	0	602	76.24	47.45	0	150	871.8	0.5

Quantity of biopsies (area 1), Quantity of days of hospital admissions (area 2), Quantity of imaging (area 3), Quantity of external consultations (area 4), Quantity of transfusion packs (area 5), Quantity of radiotherapy sessions (area 6), Quantity of preparation for radiotherapy sessions (area 7), Quantity of chemotherapy drugs (area 8), Unit cost of biopsies (area 9), Unit cost of days of hospital admissions (area 10), Unit cost of imaging (area 11), Unit cost of external consultations (area 12), Unit cost of transfusion packs (area 13), Unit cost of radiotherapy sessions (area 14), Unit cost of preparation for radiotherapy sessions (area 15), Unit cost of chemotherapy drugs (area 16).

### Cluster analysis

**Figure 2:**
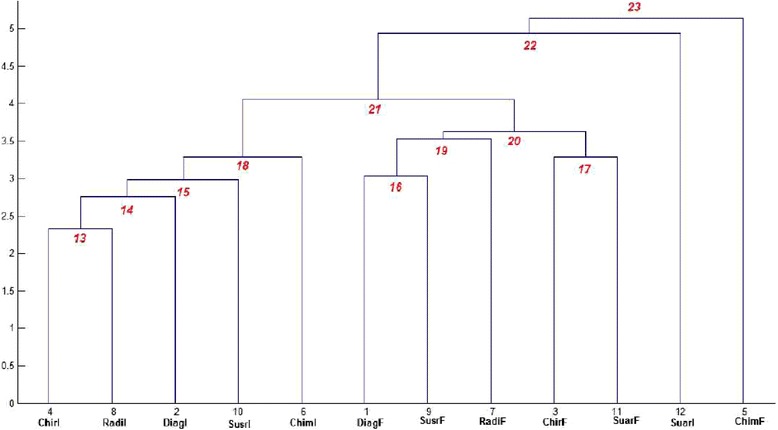
**Cluster analysis: hierarchical tree of 12 objects and newly obtained clusters (numbered from 13 to 23).** The numbers along the horizontal axis represent the indices of the objects in the original data set (1: diagnosis in France (object 1), 2: diagnosis in Italy (object 2), 3: surgery in France (object 3), 4: surgery in Italy (object 4), 5: chemotherapy in France (object 5), 6: chemotherapy in Italy (object 6), 7: radiotherapy in France (object 7), 8: radiotherapy in Italy (object 8), 9: follow-up without relapse in France (object 9), 10: follow-up without relapse in Italy (object 10), 11: follow-up with relapse in France (object 11), 12: follow-up with relapse in Italy (object 12). The newly clusters obtained by cluster analysis are numbered from 13 to 23.

As shown in Additional file 1, the best cophenetic correlation coefficient was obtained with the Euclidean metric and the Average linkage (0.83). The hierarchical tree information was shown in Figure 2, where the new clusters that were obtained by cluster analysis are numbered from 13 to 23. In Figure 2, the numbers along the horizontal axis represent the indices of the objects in the original data set. The links between objects are represented as upside down U-shaped lines, with the height of the U indicates corresponding to the distance between the objects. The analysis shows 4 clusters:

1. cluster 5 only chemotherapy in France (object 5);
2. cluster 12 only follow-up with relapse in Italy (object 12);
3. cluster 18 including surgery in Italy (object 4), radiotherapy in Italy (object 8), diagnosis in Italy (object 2), follow-up without relapse in Italy (object 10), and chemotherapy in Italy (object 6);
4. cluster 20 including diagnosis in France (object 1), follow-up without relapse in France (object 9), radiotherapy in France (object 7), surgery in France (object 3), and follow-up with relapse in France (object 11).

The details of the linkage information, including identification and specification of the pair of objects that had been linked and the distances between these objects, are shown in Additional file 5.

The optimal number of clusters based on the use of RSQ, SPRSQ, RMSSTD and the pF is shown in Figure 1. According to the pseudo-F statistics, the best choice was 4 clusters, which confirmed our interpretation based on the hierarchical tree.

#### Principal component analysis

**Figure 3:**
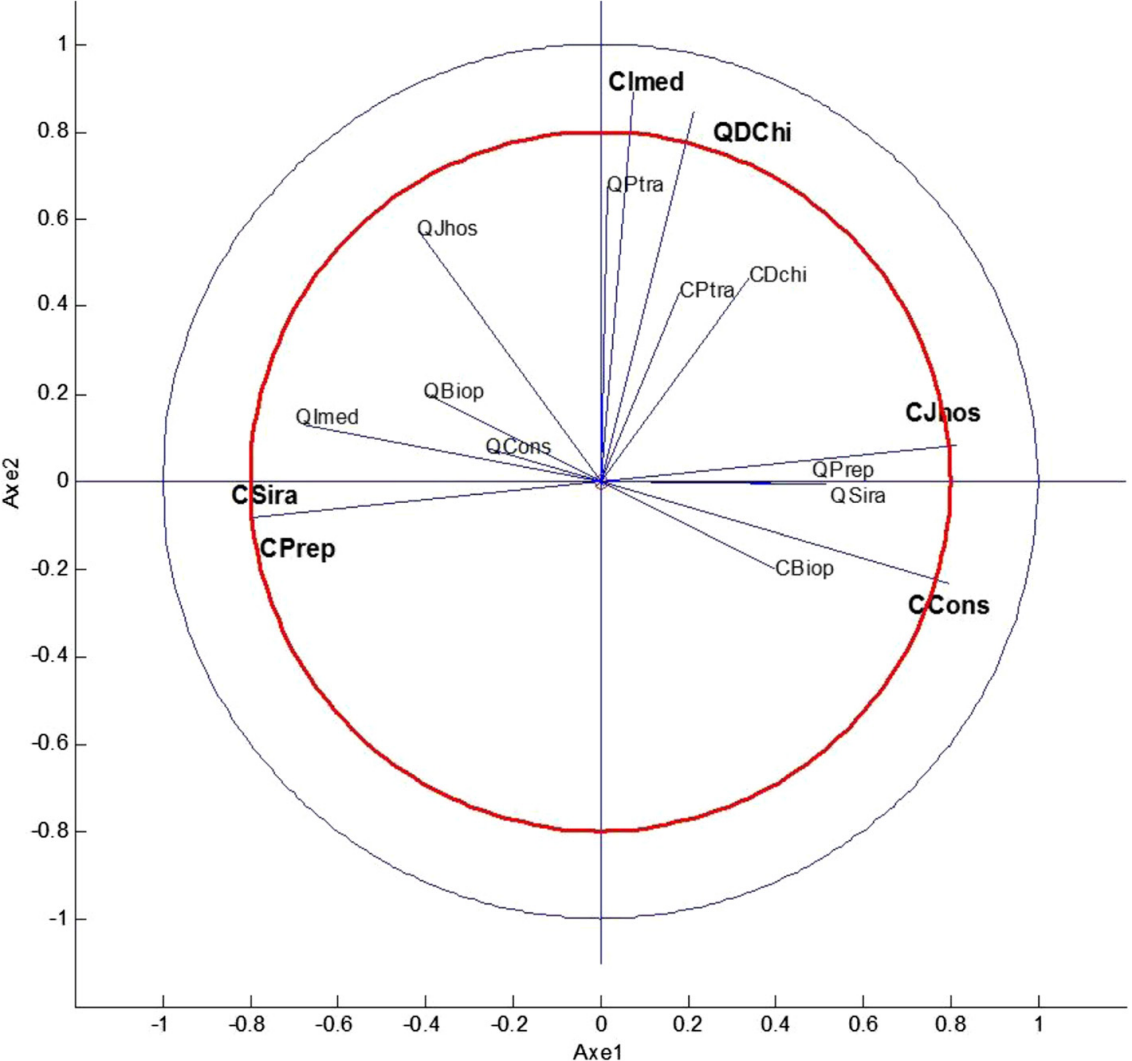
**Principal component analysis: projection of the 16 areas of variability in the factorial plane.** QBiop: Quantity of biopsies (area 1); QJhos: Quantity of days of hospital admissions (area 2); QImed: Quantity of imaging (area 3); QCons: Quantity of external consultations (area 4); QPtra: Quantity of transfusion packs (area 5); QSira: Quantity of radiotherapy sessions (area 6); QPrep: Quantity of preparation for radiotherapy sessions (area 7); QDchi: Quantity of chemotherapy drugs (area 8); CBiop: Unit cost of biopsies (area 9); CJhos: Unit cost of days of hospital admissions (area 10); CImed: Unit cost of imaging (area 11); CCons: Unit cost of external consultations (area 12); CPtra: Unit cost of transfusion packs (area 13); CSira: Unit cost of radiotherapy sessions (area 14); CPrep: Unit cost of preparation for radiotherapy sessions (area 15); CDchi: Unit cost of chemotherapy drugs (area 16).

Figure 3 shows the areas of variability (variables) in the correlation circle. The red circle of radius 0.8 drawn in the unit circle corresponds to the calculation of *r*_*j*_ given in Additional file 6. This facilitates the identification of variables. Moreover we used ***CTR***_1_(***j***) and ***CTR***_2_(***j***) (Additional file 3) and $co{s}_1^2(j)$, $co{s}_2^2(j)$ (Additional file 7). Hence, referring to the previous graph one clearly characterize several groups for variables. Along axis 1, a group on the right including *Unit cost of days of hospital admissions* (area 10) and *Unit cost of external consultations* (area 12) can be identified, as well as another group on the left comprising *Unit cost of radiotherapy sessions* (area 14) and *Unit cost of preparation for radiotherapy sessions* (area 15). Along axis 2 there is only one group (on the top) that contains *Quantity of chemotherapy drugs* (area 8) and *Unit cost of imaging* (area 11). Other areas of variability (e.g. quantity of external consultations (area 4)) were too close to the center to be interpretable.

**Figure 4:**
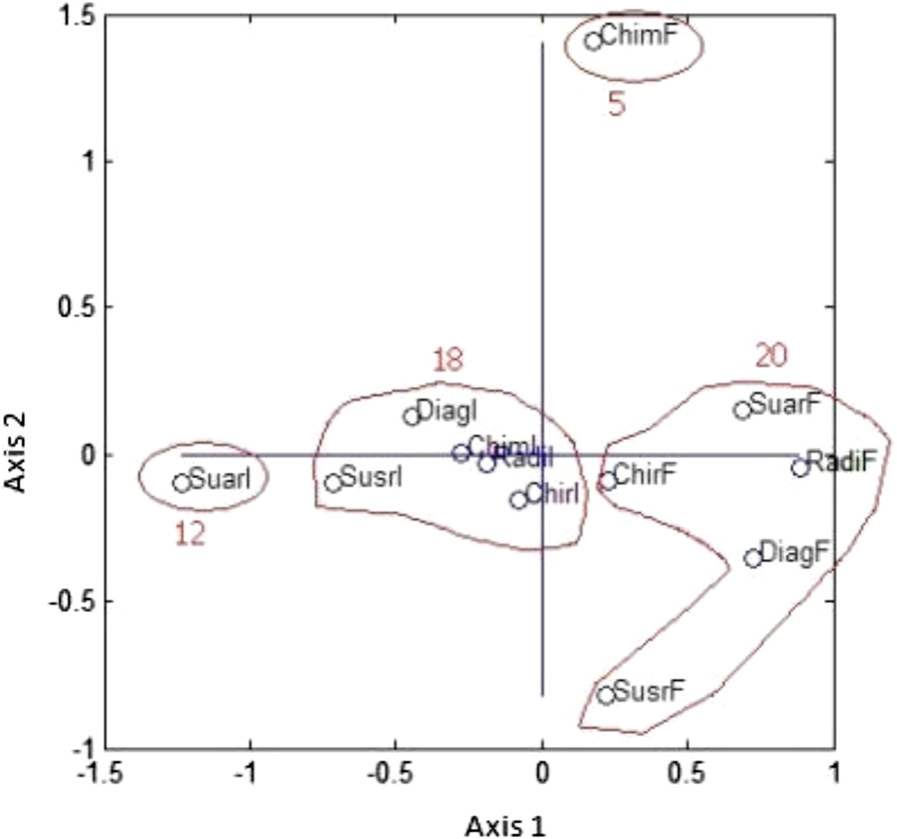
**Principal component analysis: projection of the 12 objects on the map formed by the first two components.** DiagF: diagnosis in France (object 1); DiagI: diagnosis in Italy (object 2); ChirF: surgery in France (object 3); ChirI: surgery in Italy (object 4); ChimF: chemotherapy in France (object 5); ChimI: chemotherapy in Italy (object 6); RadF: radiotherapy in France (object 7); RadI: radiotherapy in Italy (object 8); SusrF: follow-up without relapse in France (object 9); SusrI: follow-up without relapse in Italy (object 10); SuarF: follow-up with relapse in France (object 11); SuarI: follow-up with relapse in Italy (object 12); Clusters 5, 12, 18, and 20.

Figure 4 shows the projection of 12 objects on the map formed by the first two principal components, revealing four groups corresponding to the previously identified clusters. PCA also indicates that axis 1 opposes follow-up with relapse in Italy (object 12 in left-hand side) with follow-up with relapse in France (object 11 in right-hand side). The projection of 12 objects on the map formed by the first three principal components is shown in Additional file 8. The inertia of the first three components corresponded to 59.43% of the total inertia instead of 44.76% for the two first components.

In addition, an analysis that takes into account the PCA distributions of both areas of variability and objects shows, as previous evidenced, an opposition between follow-up with relapse in France (object 11) and follow-up with relapse in Italy (object 12). Therefore, it is evident that this difference mainly results from unit cost of days of hospital admissions (area 10), unit cost of external consultations (area 12), unit cost of radiotherapy sessions (area 14), and unit cost of preparation for radiotherapy sessions (area 15). Moreover, a discrepancy is observed between diagnosis in Italy (object 2) and diagnosis in France (object 1), explaining that this difference is mostly due to quantity of biopsies (area 1) and unit cost of biopsies (area 9).

Even though we utilized the first two principle axes, we tried to go further. The initial table was of the size (*n*× *m*) = (12×16). We formed the matrix **X** of standardized data and diagonalized the correlation matrix **Γ** = **X**^*T*^ × **X**. In effect, we had to find 16 eigenvalues. Based on the Kaiser criterion*,* we retained the principle components corresponding to eigenvalues above 1. In doing so, we had to retain 6 eigenvalues. Unfortunately, we can perform graphic representation in **R**^3^ at the maximum.

#### Hierarchical clustering on principal component

**Figure 5:**
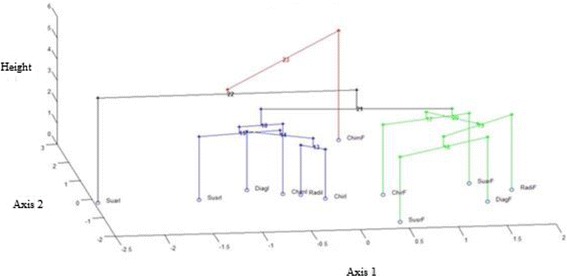
**Hierarchical clustering on principal components: tree represented on the map induced by the first two principal components.** DiagF: diagnosis in France (object 1); DiagI: diagnosis in Italy (object 2); ChirF: surgery in France (object 3); ChirI: surgery in Italy (object 4); ChimF: chemotherapy in France (object 5); ChimI: chemotherapy in Italy (object 6); RadF: radiotherapy in France (object 7); RadI: radiotherapy in Italy (object 8); SusrF: follow-up without relapse in France (object 9); SusrI: follow-up without relapse in Italy (object 10); SuarF: follow-up with relapse in France (object 11); SuarI: follow-up with relapse in Italy (object 12).

Figure 5 displays the 3-Dimensional representation of the hierarchical tree on the map produced by the first two principal components. The map shows that the four clusters are well separated on the first two principal components. Also, this graph enables visualization of the complementarity of the two methods.

Taken together, using this methodology, we were able to identify objects within our analysis that displayed high variability (i.e. lower transferability), which allowed us to distinguish areas that contributed to cost evaluation discrepancies. This study utilized both CA and PCA in order to evaluate the transferability of the results of a health economics evaluation between two countries.

## Discussion

### Discussion of results on variability in sarcoma management

Based on quantities of resources used and unit costs, the present study reveals a high discrepancy between France and Italy even though both countries reached a consensus in their clinical practice guidelines relating to all phases of sarcoma management (initial examination and diagnosis, histopathological report, surgery, chemotherapy, and radiation therapy), excluding follow-up after therapy [33,34]. Differences in the quantity of resources used could be controlled through study design (e.g. multicenter randomized trials focused on economic evaluations). However, this was not possible in this study because data were retrospectively collected and were not obtained as part of a clinical trial dedicated to this question [20].

This study also showed differences in diagnosis between Italy and France, and this heterogeneity could be explained, according to PCA, by differences in unit cost of biopsies and in quantity of biopsies. The latter could be explained by Italy employing cytology biopsies before surgical ones. This difference in management, and also difference in costs, does not permit a consistent external validity of health economic evaluations in this phase of sarcoma management. The difference between follow-up with relapse in France and in Italy was explained by a difference in unit costs, which generally have a low transferability level compared to other data elements [35]. Differences in payment systems and incentives between both countries could be valid reasons for variability, especially between clusters 5, 12, 18, and 20 as stated by Barbieri [36]. In particular, object 5 in PCA analysis and cluster 23 in CA (chemotherapy in France) are explained by the higher cost of chemotherapy in France than in Italy, as evidenced also in Table 3. This could be due to efforts by the Region Veneto Health Directorate to increase economy and efficiency in the use of resources through a number of actions, for example, defining a sort of ‘threshold’ of appropriateness of chemotherapy provided through inpatient hospitalizations to promote the outpatient health care system [37] or a deep health technology assessment for high cost imaging (for example, PET) to offer examination only when appropriate [38].

As clinicians commonly have limited personal experience managing sarcoma outside of centers of excellence (due to rarity of the disease, variety of histological types, little graduate or post-graduate medical training, etc.), it might be interesting to analyze, using CA and PCA, how clinicians’ adherence versus non-adherence to practice guidelines can modify the hierarchical structure [39-41]. In this regard, based on six phases of management (diagnosis, surgery, chemotherapy, radiotherapy, follow-up with relapse, and follow-up without relapse), two regions (Veneto and Rhône-Alpes), and compliance (or not) with clinical practice guidelines, we could generated 24 objects (6 × 2 × 2 = 24): compliant diagnosis in France (object 1); non-compliant diagnosis in France (object 2), etc. Those investigations should permit an even more precise assessment of barriers to the transferability of cost evaluations in this healthcare setting.

#### Discussion on new methodological approach to identify variability

The approach used is analytical, identifying the factors of variability and gathering them into homogeneous categories, thus making it possible to measure proximities either between objects (CA and PCA approaches) or between objects and areas of variability (PCA approach). The identification of the factors of variability and their regrouping into homogeneous categories (i.e. areas of variability) has already been studied in the literature. For example, the review of the literature carried out by Sculpher et al. shows that four groups are generally retained as the area of variability: the characteristics of the patients, the clinical parameters, the healthcare systems, and the socio-economic aspects [4].

CA and PCA have already been used in the field of health economics [42-44]. However, measurement using formal statistical methods (CA and PCA), based on the unit costs and quantities of resources used during sarcoma management, has not been performed in the past to assess variability of data in cost evaluations. CA attempts to gain first order knowledge by partitioning data points into disjoint groups based on similarity, with dissimilar data points belonging to distinct clusters. Alternatively, PCA attempts to transform high dimensional data into lower dimensional data where coherent patterns can be detected more clearly [30]. CA and PCA were found to be very complementary tools to assess transferability of health cost evaluation across locations (Figure 5); especially since in this case Matrix **A** was a sparse matrix. Moreover, the two methods present good concordance. Recent research on data mining has demonstrated the possibility of presenting both methods simultaneously representing the 3-Dimensional hierarchical tree [32]. Methods to assess the transferability of economic data are increasingly needed as the demand for economic evaluations across multiple countries often outstrips the availability of local data to support these evaluations.

#### Limitations of the study

Further research involving the application of CA and PCA to the assessment of micro-cost datasets is needed. It would be interesting to analyze the differences in cost evaluation and resources used for a single subtype of sarcoma histology or for the management of other more frequent cancers. This study only takes into account resources used and unit costs. In the future, it will be necessary to test this methodology with additional data elements, such as baseline risk, treatment effect, and health utilities in order to continue to assess the transferability of economics evaluations across locations. Potential towards health economics evaluations (e.g. multinational cost-effectiveness analysis) which are different and more complex in comparison with cost studies hasn’t been taken into account in the present study.

## Conclusions

CA and PCA provided a description of the variability in health cost evaluations between France and Italy. Indeed, using CA and PCA revealed the large spectrum of heterogeneity in sarcoma management. In future studies, regression methods could be applied after these methods to elucidate the determinants of the differences found with these analyses.

## Additional files

Additional file 1:
**The values c of the cophenetic correlation coefficient according to metric and linkage methods.**
Additional file 2:
**The values of the** ***CTR***_***α***_ **(** ***i*** **) for the objects (individuals).**
Additional file 3:
**The values of the** ***CTR***_***α***_ **(** ***j*** **) for the areas of variability (variables).**
Additional file 4:
**The values of the standardized variables Matrix** **X.**
Additional file 5:
**Linkage information, new clusters, and distances obtained by cluster analysis.**
Additional file 6:
**Distances of point-variables in the circle of correlations.**
Additional file 7:
**Quality of the representation of the variable** ***j*** **by its projection on the axis** ***α.***
Additional file 8:
**The projection of the 12 objects onto the map formed by the first three dimensions of space.**

## Abbreviations

CA: Cluster analysis
CONTICANET: Connective tissues cancers network
PCA: Principal component analysis
pF: pseudo-F statistic
RMSSTD: The root-mean-square standard deviation
RSQ: R-Square
SPRSQ: The semi-partial R-squared

## References

[1] Nixon J, Rice S, Drummond M, Boulenger S, Ulmann P, de Pouvourville G (2009). Guidelines for completing the EURONHEED transferability information checklists. Eur J Health Econ.

[2] Eldessouki R, Smith MD (2012). Health care system information sharing: a step toward better health globally. Value Health Regional Issues.

[3] Drummond M, Manca A, Sculpher M (2005). Increasing the transferability of economic evaluations: recommendations for the design, analysis, and reporting of studies. Int J Technol Assess Health Care.

[4] Sculpher MJ, Pang FS, Manca A, Drummond MF, Golder S, Urdahl H, Davies LM, Eastwood A (2004). Generalisability in economic evaluation studies in healthcare: a review and case studies. Health Technol Assess.

[5] Drummond MF, Bloom BS, Carrin G, Hillman AL, Hutchings HC, Knill-Jones RP, de Pouvourville G, Torfs K (1992). Issues in the cross-national assessment of health technology. Int J Technol Assess Health Care.

[6] Goeree R, He J, O’Reilly D, Tarride JE, Xie F, Lim M, Burke N (2011). Transferability of health technology assessments and economic evaluations: a systematic review of approaches for assessment and application. Clinicoecon Outcomes Res.

[7] Drummond M, Barbieri M, Cook J, Glick HA, Lis J, Malik F, Reed SD, Rutten F, Sculpher M, Severens J (2009). Transferability of economic evaluations across jurisdictions: ISPOR good research practices task force report. Value Health.

[8] Manca A, Sculpher MJ, Goeree R (2010). The analysis of multinational cost-effectiveness data for reimbursement decisions: a critical appraisal of recent methodological developments. Pharmacoeconomics.

[9] Rice N, Jones A (1997). Multilevel models and health economics. Health Econ.

[10] Grieve R, Nixon R, Thompson SG, Normand C (2005). Using multilevel models for assessing the variability of multinational resource use and cost data. Health Econ.

[11] Grieve R, Nixon R, Thompson SG, Cairns J (2007). Multilevel models for estimating incremental net benefits in multinational studies. Health Econ.

[12] Pinto EM, Willan AR, O’Brien BJ (2005). Cost-effectiveness analysis for multinational clinical trials. Stat Med.

[13] Willan AR, Pinto EM, O’Brien BJ, Kaul P, Goeree R, Lynd L, Armstrong PW (2005). Country specific cost comparisons from multinational clinical trials using empirical Bayesian shrinkage estimation: the Canadian ASSENT-3 economic analysis. Health Econ.

[14] Manca A, Lambert PC, Sculpher M, Rice N (2007). Cost-effectiveness analysis using data from multinational trials: the use of bivariate hierarchical modeling. Med Decis Making.

[15] Thompson SG, Nixon RM, Grieve R (2006). Addressing the issues that arise in analysing multicentre cost data, with application to a multinational study. J Health Econ.

[16] Pang F (1999). The application of multilevel modeling and cluster analysis to multinational economic evaluation data [abstract]. Value Health.

[17] Perrier L, Buja A, Mastrangelo G, Vecchiato A, Sandonà P, Ducimetière F, Blay JY, Gilly FN, Siani C, Biron P, Ranchère-Vince D, Decouvelaere AV, Thiesse P, Bergeron C, Dei Tos AP, Coindre JM, Rossi CR, Ray-Coquard I (2012). Clinicians’ adherence versus non adherence to practice guidelines in the management of patients with sarcoma: a cost-effectiveness assessment in two European regions. BMC Health Serv Res.

[18] Haute autorité de santé (HAS): *Choix Méthodologiques Pour l'évaluation économique à la HAS.* [http://www.has-sante.fr/portail/upload/docs/application/pdf/2011-11/guide_methodo_vf.pdf]

[19] Ray-Coquard I, Montesco MC, Coindre JM, Dei Tos AP, Lurkin A, Ranchère-Vince D, Vecchiato A, Decouvelaere AV, Mathoulin-Pélissier S, Albert S, Cousin P, Cellier D, Toffolatti L, Rossi CR, Blay JY, Conticanet group (2012). Sarcoma: concordance between initial diagnosis and centralized expert review in a population-based study within three European regions. Ann Oncol.

[20] Mastrangelo G, Fadda E, Cegolon L, Montesco MC, Ray-Coquard I, Buja A, Fedeli U, Frasson A, Spolaore P, Rossi CR (2010). A European project on incidence, treatment, and outcome of sarcoma. BMC Public Health.

[21] Welte R, Feenstra T, Jager H, Leidl R (2004). A decision chart for assessing and improving the transferability of economic evaluation results between countries. Pharmacoeconomics.

[22] Manca A, Rice N, Sculpher MJ, Briggs AH (2005). Assessing generasibility by location in trial-based cost-effectiveness analysis: the use of multilevel models. Health Econ.

[23] Barbieri M, Drummond M, Willke R, Chancellor J, Jolain B, Towse A (2005). Variability of cost-effectiveness estimates for pharmaceuticals in Western Europe: lessons for inferring. Value Health.

[24] Birch S, Gafni A (2003). Economics and the evaluation of health care programmes: generalisability of methods and implications for generalisability of results. Health Policy.

[25] Breiman L, Friedman J, Olshen R, Stone C (1993). Classification and Regression Trees.

[26] Cox TF, Cox MA (2001). Multidimensional Scaling.

[27] *Statistics toolbox. The Math Works, Inc. User’s Guide (Version 3)*; 2000.

[28] Halkidi M, Batistakis Y, Vazirgiannis M (2001). On clustering validation techniques. J Intell Inform Syst.

[29] Subhash S, Kumar A, Sage Publications; Inc (2006). Cluster Analysis and Factor Analysis. The Handbook of Marketing Research, Chapter 18.

[30] Jolliffe I (2010). Principal Component Analysis.

[31] Jackson JE (2003). A User’s Guide to Principal Components.

[32] Husson F, Pagès JJ: *Principal component methods, hierarchical clustering, partitional clustering: why would we need to choose for visualizing data?* [http://factominer.free.fr/docs/HCPC_husson_josse.pdf]

[33] Glickman SW, Boulding W, Roos JM, Staelin R, Peterson ED, Schulman KA (2009). Alternative pay-for-performance scoring methods: implications for quality improvement and patient outcomes. Med Care.

[34] Latry P, Martin-Latry K, Labat A e, Molimard M, Peter C (2011). Use of principal component analysis in the evaluation of adherence to statin treatment: a method to determine a potential target population for public health intervention. Fundam Clin Pharmacol.

[35] Berdeaux G, Viala M, Roborel De Climens A, Arnould B (2008). Patient-reported benefit of ReSTOR® multi-focal intraocular lenses after cataract surgery: results of principal component analysis on clinical trial data. Health Qual Life Outcomes.

[36] Barbieri M, Drummond M, Rutten F, Cook J, Glick HA, Lis J, Reed SD, Sculpher M, Severens JL, ISPOR Good Research Practices Economic DataTransferability Task Force (2010). What do international pharmacoeconomics guidelines say about economic data transferability?. Value Health.

[37] Burn: *37 del 17/04/2007,(Codice interno: 196263) Deliberazione della giunta regionale n°734 del 20 marzo.* Prestazioni di chemioterapia e radioterapia; 2007.

[38] I quaderni dell’ARSS del Veneto: *Totomographia ad emissione di positroni (PET): Valutazione del fabbisogno e piano di investimento per la Regione Veneto.* Rapporto di Health Technology Assessement; [http://www2.arssveneto.it/html_pages/documents/Quaderno_3.pdf]

[39] Stiller CA, Trama A, Serraino D, Rossi S, Navarro C, Chirlaque MD, Casali PG, RARECARE Working Group (2013). Descriptive epidemiology of sarcomas in Europe: report from the rarecare project. Eur J Cancer.

[40] FNCLCC (1995). Standards, Options et Recommandations pour la prise en charge des patients adultes atteints de sarcome des tissus mous, de sarcome utérin ou de tumeur stromale gastro-intestinale.

[41] *Italian National Research Council in Italy.* [http://progettooncologia.cnr.it/bridge/attivita-direzione.html]

[42] Guo JJ, Jing Y, Nguyen K, Fan H e, Kelton CM (2008). Principal components analysis of drug expenditure and utilisation trends for major therapeutic classes in US Medicaid programmes. J Med Econ.

[43] Holmes GM, Pink GH (2012). Adoption and perceived effectiveness of financial improvement strategies in critical access hospitals. J Rural Health.

[44] Ding C, He X (2004). K-Means Clustering Via Principal Component Analysis. Proceeding ICML’04: Proceedings of the 21th International Conference on Machine Learning.

