# Supplementary material for: Transferability of health cost evaluation across locations in oncology: cluster and principal component analysis as an explorative tool
Source: BMC Health Serv Res. 2014 Nov 18;14:537. doi: 10.1186/s12913-014-0537-x (PMC4241216; doi:10.1186/s12913-014-0537-x)
Supplement: Additional file 1: — The values c of the cophenetic correlation coefficient according to metric and linkage methods. [file 12913_2014_537_MOESM1_ESM.docx]

Additional file 1. The values c of the cophenetic correlation coefficient according to metric and linkage methods

| \|  \| Metric \| \| --- \| --- \| \| Linkage method \|  \| | Euclidean | Mahalanobis | City Block |
| --- | --- | --- | --- | --- | --- | --- | --- |
| Single | 0.75975 | 0.58138 | 0.53069 |
| Complete | 0.63780 | 0.54648 | 0.54711 |
| Average | 0.83035 | 0.66621 | 0.64108 |
| Centroid | 0.77448 | 0.65604 | 0.70065 |
| Ward | 0.62363 | 0.47693 | 0.53891 |
