# Supplementary material for: Transferability of health cost evaluation across locations in oncology: cluster and principal component analysis as an explorative tool
Source: BMC Health Serv Res. 2014 Nov 18;14:537. doi: 10.1186/s12913-014-0537-x (PMC4241216; doi:10.1186/s12913-014-0537-x)
Supplement: Additional file 2: — The values of the CTR α ( i ) for the objects (individuals). [file 12913_2014_537_MOESM2_ESM.docx]

Additional file 2. The values of the ${CTR}_{\text{α}}\left( i \right)$ for the objects (individuals)

The contribution of the observation *i* to component α is denoted ${CTR}_{\text{α}}\left( i \right)$ obtained as ${CTR}_{\alpha}\left( i \right)=\frac{\psi_{\alpha}^{2}\left( i \right)}{\sum_{i} \psi_{\alpha}^{2}\left( i \right)}$ with $\Psi_{\alpha}^{2}\left( i \right)$ the square of the coordinate of individual-point *i.*

| Objects | $\boldsymbol{CTR}_{\text{1}}\left( \boldsymbol{i} \right)$ | $\boldsymbol{CTR}_{\text{2}}\left( \boldsymbol{i} \right)$ | $\boldsymbol{CTR}_{\text{3}}\left( \boldsymbol{i} \right)$ |
| --- | --- | --- | --- |
| Diagnosis in France (object 1) | 0.1237 | 0.0435 | 0.24317 |
| Diagnosis in Italy (object 2) | 0.0464 | 0.0057 | 0.3915 |
| Surgery in France (object 3) | 0.0119 | 0.0029 | 0.0010 |
| Surgery in Italy (object 4) | 0.0014 | 0.0084 | 0.0111 |
| Chemotherapy in France (object 5) | 0.0072 | 0.6888 | 0.0548 |
| Chemotherapy in Italy (object 6) | 0.0177 | 0.0000 | 0.0147 |
| Radiotherapy in France (object 7) | 0.1844 | 0.0007 | 0.1371 |
| Radiotherapy in Italy (object 8) | 0.0083 | 0.0003 | 0.0199 |
| Follow-up without relapse in France (object 9) | 0.0118 | 0.2353 | 0.0077 |
| Follow-up without relapse in Italy (object 10) | 0.1197 | 0.0031 | 0.0249 |
| Follow-up with relapse in France (object 11) | 0.1122 | 0.0079 | 0.0034 |
| Follow-up with relapse in Italy (object 12) | 0.3554 | 0.0035 | 0.0910 |
