# Supplementary material for: Transferability of health cost evaluation across locations in oncology: cluster and principal component analysis as an explorative tool
Source: BMC Health Serv Res. 2014 Nov 18;14:537. doi: 10.1186/s12913-014-0537-x (PMC4241216; doi:10.1186/s12913-014-0537-x)
Supplement: Additional file 3: — The values of the CTR α ( j ) for the areas of variability (variables). [file 12913_2014_537_MOESM3_ESM.docx]

Additional file 3. The values of the ${CTR}_{\text{α}}\left( j \right)$ for the areas of variability (variables)

The contribution of the variable *j* to component α is denoted ${CTR}_{\text{α}}\left( j \right)$ obtained as ${CTR}_{\alpha}\left( j \right)=\frac{\varphi_{\alpha}^{2}\left( i \right)}{\sum_{i} \varphi_{\alpha}^{2}\left( i \right)}$ with $\varphi_{\alpha}^{2}\left( i \right)$ the square of the coordinate of variable-point *j.*

| Areas of variability | $\boldsymbol{CTR}_{\text{1}}\left( \boldsymbol{j} \right)$ | $\boldsymbol{CTR}_{\text{2}}\left( \boldsymbol{j} \right)$ | $\boldsymbol{CTR}_{\text{3}}\left( \boldsymbol{j} \right)$ |
| --- | --- | --- | --- |
| Q. of biopsies (area 1) | 0.0376 | 0.0140 | 0.2667 |
| Q. of days of hospitalization (area 2) | 0.0409 | 0.1147 | 0.0099 |
| Q. of imaging (area 3) | 0.1083 | 0.0057 | 0.0083 |
| Q. of external consultations (area 4) | 0.0158 | 0.0019 | 0.0169 |
| Q. of transfusion packs (area 5) | <0.0001 | 0.1585 | 0.0139 |
| Q. of radiotherapy sessions (area 6) | 0.0624 | <0.0001 | 0.1064 |
| Q. of preparation for radiotherapy sessions (area 7) | 0.0487 | <0.0001 | 0.0969 |
| Q. of chemotherapy drugs (area 8) | 0.0107 | 0.2502 | 0.0011 |
| Unit cost of biopsies (area 9) | 0.0376 | 0.0140 | 0.2667 |
| Unit cost of days of hospital admissions (area 10) | 0.1568 | 0.0024 | 0.0405 |
| Unit cost of imaging (area 11) | 0.0013 | 0.2751 | 0.0182 |
| Unit cost of external consultations (area 12) | 0.1486 | 0.0185 | 0.0461 |
| Unit cost of transfusion packs (area 13) | 0.0078 | 0.0649 | 0.0258 |
| Unit cost of radiotherapy sessions (area 14) | 0.1481 | 0.0023 | 0.0351 |
| Unit cost of preparation for radiotherapy sessions (area 15) | 0.1481 | 0.0023 | 0.0351 |
| Unit cost of chemotherapy drugs (area 16) | 0.0274 | 0.0754 | 0.0124 |

Q.= Quantity.
