# Supplementary material for: Transferability of health cost evaluation across locations in oncology: cluster and principal component analysis as an explorative tool
Source: BMC Health Serv Res. 2014 Nov 18;14:537. doi: 10.1186/s12913-014-0537-x (PMC4241216; doi:10.1186/s12913-014-0537-x)
Supplement: Additional file 4: — The values of the standardized variables Matrix X. [file 12913_2014_537_MOESM4_ESM.docx]

Additional file 4. The values of the standardized variables Matrix **X**

|  | Areas of variability | | | | | | | | | | | | | | | | |
| --- | --- | --- | --- | --- | --- | --- | --- | --- | --- | --- | --- | --- | --- | --- | --- | --- | --- |
| Objects | Area 1  Q. Biopsies | Area 2  Q. days of hospitalization | Area 3  Q. imaging | Area 4  Q. external consultations | Area 5  Q. transfusions | Area 6  Q. rad. sessions | Area 7  Q. rad. preparation | Area 8  Q. chemotherapy drugs | Area 9  C. biopsy | Area 10  C. days of hospitalization | Area 11  C. imaging | Area 12  C. external consultation | Area 13  C. transfusion | Area 14  C. rad. session | Area 15  C. rad. preparation | | Area 16  C. chemotherapy drugs |
| 1. Diagnosis France | -1.00 | -1.08 | -0.43 | -1.45 | 0.00 | 0.00 | 0.00 | 0.00 | 1.00 | 1.00 | -0.02 | 1.00 | 0.00 | 0.00 | | 0.00 | 0.00 |
| 2. Diagnosis Italy | 1.00 | -0.52 | -0.09 | -1.20 | 0.00 | 0.00 | 0.00 | 0.00 | -1.00 | -1.00 | -0.03 | -1.00 | 0.00 | 0.00 | | 0.00 | 0.00 |
| 3. Surgery France | 0.00 | 0.57 | -1.03 | 0.00 | -1.23 | 0.00 | 0.00 | 0.00 | 0.00 | 1.00 | -0.11 | 0.00 | 1.00 | 0.00 | | 0.00 | 0.00 |
| 4. Surgery Italy | 0.00 | 0.18 | -0.89 | 0.00 | -0.03 | 0.00 | 0.00 | 0.00 | 0.00 | -1.00 | -0.03 | 0.00 | -1.00 | 0.00 | | 0.00 | 0.00 |
| 5. Chemotherapy France | 0.00 | 1.55 | 0.16 | 0.00 | 1.55 | 0.00 | 0.00 | 1.45 | 0.00 | 1.00 | 2.70 | 0.00 | 1.00 | 0.00 | | 0.00 | 0.28 |
| 6. Chemotherapy Italy | 0.00 | 1.25 | 1.15 | 0.00 | -0.29 | 0.00 | 0.00 | 0.62 | 0.00 | -1.00 | -1.13 | 0.00 | -1.00 | 0.00 | | 0.00 | 0.84 |
| 7. Radiotherapy France | 0.00 | -1.11 | 0.00 | 0.00 | 0.00 | 1.49 | 1.22 | 0.00 | 0.00 | 1.00 | 0.00 | 0.00 | 0.00 | -1.22 | | -1.22 | 0.00 |
| 8. Radiotherapy Italy | 0.00 | 0.25 | 0.00 | 0.00 | 0.00 | 0.90 | 1.22 | 0.00 | 0.00 | -1.00 | 0.00 | 0.00 | 0.00 | 0.82 | | 0.82 | 0.00 |
| 9. Follow-up without  relapse France | 0.00 | -1.21 | -0.56 | 0.08 | 0.00 | 0.00 | 0.00 | -1.30 | 0.00 | 1.00 | -1.19 | 1.00 | 0.00 | 0.00 | | 0.00 | -1.33 |
| 10. Follow-up without  relapse Italy | 0.00 | -1.12 | 0.16 | 0.43 | 0.00 | -0.88 | -0.92 | 0.00 | 0.00 | -1.00 | -0.03 | -1.00 | 0.00 | 0.82 | | 0.82 | 0.00 |
| 11. Follow-up with  relapse France | 0.00 | -0.24 | -0.83 | 1.10 | 0.00 | -0.66 | -0.66 | 0.13 | 0.00 | 1.00 | -0.11 | 1.00 | 0.00 | -1.22 | | -1.22 | 1.21 |
| 12. Follow-up with  relapse Italy | 0.00 | 1.48 | 2.36 | 1.03 | 0.00 | -0.86 | -0.86 | -0.90 | 0.00 | -1.00 | -0.03 | -1.00 | 0.00 | 0.82 | | 0.82 | -0.99 |

Quantity of biopsies (area 1), Quantity of days of hospital admissions (area 2), Quantity of imaging (area 3), Quantity of external consultations (area 4), Quantity of transfusion packs (area 5), Quantity of radiotherapy sessions (area 6), Quantity of preparation for radiotherapy sessions (area 7), Quantity of chemotherapy drugs (area 8), Unit cost of biopsies (area 9), Unit cost of days of hospital admissions (area 10), Unit cost of imaging (area 11), Unit cost of external consultations (area 12), Unit cost of transfusion packs (area 13), Unit cost of radiotherapy sessions (area 14), Unit cost of preparation for radiotherapy sessions (area 15), Unit cost of chemotherapy drugs (area 16).
