# Supplementary material for: Transferability of health cost evaluation across locations in oncology: cluster and principal component analysis as an explorative tool
Source: BMC Health Serv Res. 2014 Nov 18;14:537. doi: 10.1186/s12913-014-0537-x (PMC4241216; doi:10.1186/s12913-014-0537-x)
Supplement: Additional file 5: — Linkage information, new clusters, and distances obtained by cluster analysis. [file 12913_2014_537_MOESM5_ESM.docx]

Additional file 5 Linkage information, new clusters, and distances obtained by cluster analysis

| New Clusters | Object_1_ | Object_2_ | Distances |
| --- | --- | --- | --- |
| 13  (Surgery Italy+Radiotherapy Italy) | 4  (Surgery Italy) | 8  (Radiotherapy Italy) | 2.3321 |
| 14  (13+Diagnosis Italy) | 13  (Surgery Italy+Radiotherapy Italy) | 2  (Diagnosis I) | 2.7545 |
| 15  (14+Follow-up without relapse Italy) | 14  (13+Diagnosis Italy) | 10  (Follow-up without relapse Italy) | 2.9808 |
| 16  (Diagnosis France+Follow-up without relapse France) | 1  (Diagnosis France) | 9  (Follow-up without relapse France) | 3.0351 |
| 17  (Surgery in France+ Follow-up with relapse France) | 3  (Surgery in France) | 11  Follow-up with relapse France) | 3.2844 |
| 18  (15+Chemotherapy Italy) | 15  (14+Follow-up without relapse Italy) | 6  (Chemotherapy Italy) | 3.2845 |
| 19  (16+radiotherapy F) | 16  (Diagnosis France+Follow-up without relapse France) | 7  (radiotherapy F) | 3.5311 |
| 20  (19+Surgery in France+ Follow-up with relapse France) | 19  (16+radiotherapy F) | 17  (Surgery in France+ Follow-up with relapse France) | 3.6239 |
| 21  (18+20) | 18  (15+Chemotherapy Italy) | 20  (19+Surgery in France+ Follow-up with relapse France) | 4.0539 |
| 22  (21+ Follow-up with relapse Italy) | 21  (18+20) | 12  (Follow-up with relapse Italy) | 4.9372 |
| 23  (22+Chemotherapy France) | 22  (21+ Follow-up with relapse Italy) | 5  (Chemotherapy France) | 5.1436 |
