# Supplementary material for: Transferability of health cost evaluation across locations in oncology: cluster and principal component analysis as an explorative tool
Source: BMC Health Serv Res. 2014 Nov 18;14:537. doi: 10.1186/s12913-014-0537-x (PMC4241216; doi:10.1186/s12913-014-0537-x)
Supplement: Additional file 6: — Distances of point-variables in the circle of correlations. [file 12913_2014_537_MOESM6_ESM.docx]

Additional file 6. Distances of point-variables in the circle of correlations

| Areas of variability | $\phi_{1}(j)$ | $\phi_{2}(j)$ |  |
| --- | --- | --- | --- |
| Unit cost of imaging (area 11) | 0.0738 | 0.89074 | 0.8938 |
| Quantity of chemotherapy drugs (area 8) | 0.2139 | 0.8495 | 0.8760 |
| Unit cost of external consultations (area 12) | 0.7971 | -0.2309 | 0.8299 |
| Unit cost of days of hospital admissions (area 10) | 0.8190 | 0.0837 | 0.8233 |
| Unit cost of radiotherapy sessions (area 14) | -0.7959 | -0.0821 | 0.8001 |
| Unit cost of preparation for radiotherapy sessions (area 15) | -0.7959 | -0.0821 | 0.8001 |
| Quantity of days of hospital admissions (area 2) | -0.4183 | 0.5752 | 0.7112 |
| Quantity of imaging (area 3) | -0.6805 | 0.1282 | 0.6925 |
| Quantity of transfusion packs (area 5) | 0.0183 | 0.6762 | 0.6764 |
| Unit cost of chemotherapy drugs (area 16) | 0.3423 | 0.4663 | 0.5785 |
| Quantity of radiotherapy sessions (area 6) | 0.5165 | -0.0059 | 0.5165 |
| Unit cost of transfusion packs (area 13) | 0.1821 | 0.4328 | 0.4695 |
| Quantity of preparation for radiotherapy sessions (area 7) | 0.4565 | -0.0041 | 0.4565 |
| Quantity of biopsies (area 1) | -0.4010 | 0.2010 | 0.4486 |
| Unit cost of biopsies (area 9) | 0.4010 | -0.2010 | 0.4486 |
| Quantity of external consultations (area 4) | -0.259 | 0.0751 | 0.2703 |
