# Supplementary material for: Transferability of health cost evaluation across locations in oncology: cluster and principal component analysis as an explorative tool
Source: BMC Health Serv Res. 2014 Nov 18;14:537. doi: 10.1186/s12913-014-0537-x (PMC4241216; doi:10.1186/s12913-014-0537-x)
Supplement: Additional file 7: — Quality of the representation of the variable j by its projection on the axis α. [file 12913_2014_537_MOESM7_ESM.docx]

Additional file 7. Quality of the representation of the variable $j$ by its projection on the axis $\alpha$

| Areas of variability | cos₁²(j) | cos₂²(j) |
| --- | --- | --- |
| Q. of biopsies (area 1) | 0.16082 | 0.04041 |
| Q. of days of hospitalization (area 2) | 0.17495 | 0.33086 |
| Q. of imaging (area 3) | 0.46308 | 0.01644 |
| Q. of external consultations (area 4) | 0.06746 | 0.00563 |
| Q. of transfusion packs (area 5) | 0.00033 | 0.45723 |
| Q. of radiotherapy sessions (area 6) | 0.26677 | 0.00003 |
| Q. of preparation for radiotherapy sessions (area 7) | 0.20838 | 0.00002 |
| Q. of chemotherapy drugs (area 8) | 0.04577 | 0.72168 |
| Unit cost of biopsies (area 9) | 0.16082 | 0.04041 |
| Unit cost of days of hospital admissions (area 10) | 0.67083 | 0.00701 |
| Unit cost of imaging (area 11) | 0.00544 | 0.79342 |
| Unit cost of external consultations (area 12) | 0.63545 | 0.05332 |
| Unit cost of transfusion packs (area 13) | 0.03316 | 0.18729 |
| Unit cost of radiotherapy sessions (area 14) | 0.63348 | 0.00675 |
| Unit cost of preparation for radiotherapy sessions (area 15) | 0.63348 | 0.00675 |
| Unit cost of chemotherapy drugs (area 16) | 0.11717 | 0.21742 |

Q.= Quantity.
