# Supplementary material for: Transferability of health cost evaluation across locations in oncology: cluster and principal component analysis as an explorative tool
Source: BMC Health Serv Res. 2014 Nov 18;14:537. doi: 10.1186/s12913-014-0537-x (PMC4241216; doi:10.1186/s12913-014-0537-x)
Supplement: Additional file 8: — The projection of the 12 objects onto the map formed by the first three dimensions of space. [file 12913_2014_537_MOESM8_ESM.docx]

Additional file 8. The projection of the 12 objects onto the map formed by the first three dimensions of space


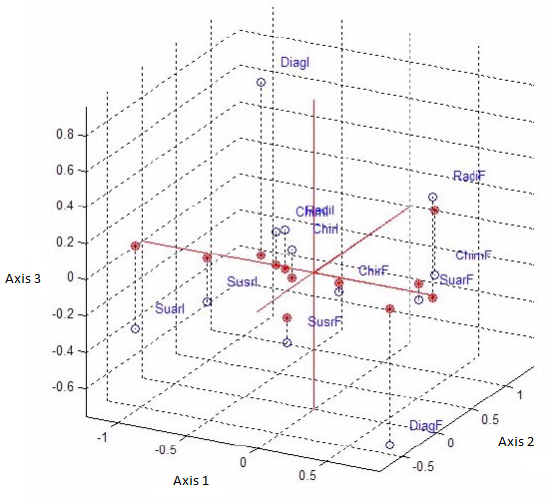


DiagF: diagnosis in France (object 1); DiagI: diagnosis in Italy (object 2); ChirF: surgery in France (object 3); ChirI: surgery in Italy (object 4); ChimF: chemotherapy in France (object 5); ChimI: chemotherapy in Italy (object 6); RadF: radiotherapy in France (object 7); RadI: radiotherapy in Italy (object 8); SusrF: follow-up without relapse in France (object 9); SusrI: follow-up without relapse in Italy (object 10); SuarF: follow-up with relapse in France (object 11); SuarI: follow-up with relapse in Italy (object 12).
